# matter: an R package for rapid prototyping with larger-than-memory datasets on disk

**DOI:** 10.1093/bioinformatics/btx392

**Published:** 2017-06-15

**Authors:** Kylie A Bemis, Olga Vitek

**Affiliations:** 1 College of Computer and Information Science, Northeastern University, Boston, MA, USA; 2 Department of Chemistry and Chemical Biology, College of Science, Northeastern University, Boston, MA, USA

## Abstract

**Summary:**

We introduce matter, an R package for direct interactions with larger-than-memory datasets, stored in an arbitrary number of files of any size. matter is primarily designed for datasets in new and rapidly evolving file formats, which may lack extensive software support. matter enables a wide variety of data exploration and manipulation steps and is extensible to many bioinformatics applications. It supports reproducible research by minimizing the need of converting and storing data in multiple formats. We illustrate the performance of matter in conjunction with the Bioconductor package Cardinal for analysis of high-resolution, high-throughput mass spectrometry imaging experiments.

**Availability and implementation:**

The package, vignettes and examples of applications in several areas of bioinformatics are available open-source at www.bioconductor.org under the Artistic-2.0 license.

## 1 Introduction

The need for flexible and scalable computation pervades all areas of data science, including bioinformatics. For example, rapid increases in mass and spatial resolutions of mass spectrometry imaging (MSI) experiments have led to datasets of 1–100 GB per biological replicate. The data are stored in specialized formats, e.g. imzML or Analyze, and may be spread across dozens of files. New statistical methods must be developed for these data, and the speed of method development must match the speed of technological advances.

The R programming language is the tool of choice for rapid development of statistical methodology, due to its flexibility and its substantial analytic resources, e.g. in Bioconductor. However, base R is limited to datasets that fit entirely in computer memory. Recently, R packages bigmemory (Kane *et al.*, 2013) and ff (Adler *et al.*, 2014) were designed to work with large datasets; however, they have strict requirements on file formats and structures on disk. For example, bigmemory matrices cannot be easily transposed, and ff arrays are limited to 2^31^ − 1 total elements. Both assume that the data are stored contiguously on disk in a single file, which limits the ability to create a single dataset from multiple files without further file duplication.

We introduce matter, a free and open-source R package, which provides a flexible infrastructure for datasets on disk. It is designed for direct interactions with larger-than-memory datasets in new and rapidly evolving file formats, which may not yet have extensive software support. These datasets create the biggest difficulty when working with in R, due to both format and size. matter also offers a suite of unique functionalities for well-supported formats, including efficient delayed arithmetic operations, and allowing fast data manipulation and transformation without touching a byte on disk. We illustrate the performance of matter in conjunction with the R package Cardinal (Bemis *et al.*, 2015) for analysis of MSI experiments. The package’s main vignette provides additional examples of extensions to other bioinformatics applications, and two supplementary vignettes demonstrate performance comparisons with bigmemory and ff.

## 2 Description

### 2.1 Design and data representation

matter makes no assumptions of file format or structure and is designed to access any type of uncompressed file. This is accomplished by building the virtual representation of a dataset around the concept of ‘atomic’ segments of data storage.

In matter, any uncompressed file structure is viewed as a composition of data ‘atoms’. An atom is a sequence of data elements stored contiguously on disk, which can be loaded into memory in a single read operation. An atom is defined by a file path, a data type, a byte-offset and an extent. Atoms can be composed together to form virtual vectors, matrices, arrays and data frames. A matter object is simply a sequence of metadata ‘atoms’ describing where on disk each part of a dataset can be found.

The user interacts with a matter object the same way as if the data were fully readable on disk. For example, when subsetting a matter vector, the indices of the vector are mapped to their locations on disk, and that subset of data is loaded into memory and returned to the user. After the calculation with this portion of the data completes, the memory is freed. Such reduction of memory footprint comes at the cost of heavy disk use. matter compensates for this by utilizing sequential read/writes over random read/writes whenever possible and by minimizing the total number of atomic read/write operations.

### 2.2 Data analysis requirements

When working with larger-then-memory datasets, statistical methods must operate on portions of the data at a time and produce smaller output. As a result, some frequently used algorithms must be modified. matter simplifies this by implementing common operations, e.g. arithmetic operations, matrix multiplication, memory-efficient calculations of summary statistics (e.g. row- and column-wise mean and variance for matter matrices), apply method for user-specified operations on rows and columns of on-disk matrices and transpose operations.

For statistical analyses, matter implements an interface to the biglm package (Lumley, 2013) for memory-efficient fitting of generalized linear models. matter also implements an interface to the irlba package (Baglama and Reichel, 2015) for principal components analysis (PCA) of larger-than-memory datasets. matter is not directly compatible with R code that relies on code written in C or C ++.

As an example of more comprehensive analyses, the MSI package Cardinal interfaces with matter to provide support for larger-than-memory MSI datasets. It supports preprocessing, spectral and ion image visualization and PCA. Cardinal’s batchProcess method loads one spectrum at a time using matter, and typically reduces the dataset to a size that can be fully loaded into memory and used with other tools. These example interfaces can be followed to prototype new statistical methods for larger-then-memory datasets in other areas of research.

### 2.3 Hardware and data requirements

matter is compatible with Windows, Mac and Linux operating systems. It requires R ≥3.0 and Bioconductor ≥3.4. Users can specify any custom binary file formats, provided that data elements are stored at known byte-offsets. The total available memory should be twice the largest portion of the data used in an atomic calculation. matter has been tested on datasets up to 42 GB in size.

For compatibility with R, the maximum number of data elements in vectors and matrices is limited to 2^52^, and the maximal extent of matrix dimensions is limited to 2^31^ − 1. The total number of elements in the matrix can exceed the limit on dimensions. matter runs optimally with contiguous data stored on a fast storage device, such as a solid state drive (SSD), but this is not a requirement.

## 3 Examples of performance

To evaluate the performance of matter in situations where file format is not an issue, we considered two typical analyses. Table 1 summarizes the results of linear regression and PCA on simulated datasets of 1.2 GB, on a 2012 MacBook Pro 2.6 GHz with SSD. The use of matter (as opposed to R matrices) dramatically reduced the memory footprint. matter had a smaller footprint than bigmemory and ff and performed faster than ff. Since bigmemory uses mmap to map the on-disk data to virtual memory, it performs faster on datasets that can fit into memory entirely but has a higher memory footprint.

**Table 1:** Performance of matter with bigmemory and ff for linear regression and calculation of the first two principal components on simulated datasets of 1.2 GB

Linear regression				Principle component analysis			
Method	Mem. used	Mem. overhead	Time (s)	Method	Mem. used	Mem. overhead	Time (s)
R matrices + lm	7 GB	1.4 GB	33	R matrices + svd	3.9 GB	2.4 GB	66
bigmemory + biglm	4.4 GB	3.9 GB	21	bigmemory + irlba	3.1 GB	2.7 GB	15
ff + biglm	1.9 MB	1.6 GB	57	ff + irlba	1.8 GB	1.4 GB	130
matter + biglm	1 GB	660 MB	47	matter + irlba	890 MB	490 MB	110

Memory overhead is the maximum memory used during the execution minus the memory used after completion.

Table 2 illustrates the performance of matter for all the benchmark MSI datasets in the ‘continuous’ imzML format in Oetjen *et al.* (2015). Cardinal and matter performed PCA on a personal computer in a reasonable time. These analyses are impossible with the base R infrastructure. These analyses were also impossible with ff, which currently does not support such large files. They were slower with bigmemory. The vignettes included with matter package fully compare the performance of matter to bigmemory and ff on these datasets.

**Table 2:** Calculation of the first two principal components on all the MSI datasets in ‘continuous’ imzML format in Oetjen *et al.* (2015)

Dataset	Size	Pixels	Features	Mem. used	Mem. overhead	Time
3D microbial time course	2.9 GB	17 672	40 299	228 MB	50 MB	13 min, 6 s
3D oral squamous cell carcinoma	25.4 GB	828 558	7680	977 MB	668 MB	2 h, 7 min, 9 s
3D mouse pancreas	26.4 GB	497 225	13 312	628 MB	370 MB	2 h, 12 min, 46 s
3D mouse kidney	41.8 GB	1 362 830	7680	1.5 GB	1.1 GB	3 h, 22 min, 24 s

Comparisons with bigmemory and ff on these datasets are available in the package vignettes.

## 4 Discussion

matter supports direct interactions with larger-than-memory data on disk. It is best suited for data in new and rapidly evolving file formats with known structure. Although many laboratories have adopted standard formats such as NetCDF or HDF5, new file formats such as imzML will continue to proliferate as technology evolves. matter exists in part to ensure that such data can always be accessed in R. The direct interaction with these data is advantageous because (i) it facilitates the work with new technologies that do not yet have extensive software support, (ii) it minimizes file conversion effort, memory and time and (iii) it contributes to reproducible research, as it eliminates the need of keeping track of multiple and separately stored files and minimizes loss of information that can sometimes occur during the conversion.
